# Accurate quantification of chromosomal lesions via short tandem repeat analysis using minimal amounts of DNA

**DOI:** 10.1136/jmedgenet-2017-104528

**Published:** 2017-06-09

**Authors:** Johann-Christoph Jann, Daniel Nowak, Florian Nolte, Stephanie Fey, Verena Nowak, Julia Obländer, Jovita Pressler, Iris Palme, Christina Xanthopoulos, Alice Fabarius, Uwe Platzbecker, Aristoteles Giagounidis, Katharina Götze, Anne Letsch, Detlef Haase, Richard Schlenk, Gesine Bug, Michael Lübbert, Arnold Ganser, Ulrich Germing, Claudia Haferlach, Wolf-Karsten Hofmann, Maximilian Mossner

**Affiliations:** 1 III Medizinische Klinik, Hämatologie und Onkologie, Universitätsmedizin Mannheim, Mannheim, Germany; 2 Medizinische Klinik und Poliklinik I, Universitatsklinikum Carl Gustav Carus, Dresden, Germany; 3 Klinik für Hämatologie, Onkologie und Palliativmedizin, Marienhospital, Düsseldorf, Germany; 4 III. Medizinischen Klinik des Klinikums rechts der Isar, Technische Universitat Munchen, Munchen, Germany; 5 Medizinische Klinik für Hämatologie, Onkologie, Campus Benjamin Franklin, Charite Universitatsmedizin Berlin, Berlin, Germany; 6 Klinik für Hämatologie und Medizinische Onkologie, Georg-August-Universitat Gottingen Universitatsmedizin, Gottingen, Germany; 7 NCT Trial Center, Nationales Centrum für Tumorerkrankungen (NCT), Heidelberg, Gemany; 8 Medizinische Klinik II, Abteilung für Hämatologie/Onkologie, Klinikum der Johann Wolfgang Goethe-Universitat Frankfurt, Frankfurt am Main, Germany; 9 Abteilung für Innere Medizin I, Hämatologie und Onkologie, Universitatsklinikum Freiburg, Freiburg, Germany; 10 Abteilung für Hämatologie, Hämostaseologie, Onkologie und Stammzelltransplantation, Medizinische Hochschule Hannover, Hannover, Germany; 11 Abteilung für Hämatologie, Onkologie und klinische Immunologie, Heinrich-Heine-Universitat Dusseldorf Medizinische Fakultat, Dusseldorf, Germany; 12 Münchner Leukämie Labor, München, Germany

**Keywords:** Chromosomal deletion, short tandem repeats, PCR, deletion 5q, myelodysplastic syndrome

## Abstract

**Background:**

Cytogenetic aberrations such as deletion of chromosome 5q (del(5q)) represent key elements in routine clinical diagnostics of haematological malignancies. Currently established methods such as metaphase cytogenetics, FISH or array-based approaches have limitations due to their dependency on viable cells, high costs or semi-quantitative nature. Importantly, they cannot be used on low abundance DNA. We therefore aimed to establish a robust and quantitative technique that overcomes these shortcomings.

**Methods:**

For precise determination of del(5q) cell fractions, we developed an inexpensive multiplex-PCR assay requiring only nanograms of DNA that simultaneously measures allelic imbalances of 12 independent short tandem repeat markers.

**Results:**

Application of this method to n=1142 samples from n=260 individuals revealed strong intermarker concordance (R²=0.77–0.97) and reproducibility (mean SD: 1.7%). Notably, the assay showed accurate quantification via standard curve assessment (R²>0.99) and high concordance with paired FISH measurements (R²=0.92) even with subnanogram amounts of DNA. Moreover, cytogenetic response was reliably confirmed in del(5q) patients with myelodysplastic syndromes treated with lenalidomide. While the assay demonstrated good diagnostic accuracy in receiver operating characteristic analysis (area under the curve: 0.97), we further observed robust correlation between bone marrow and peripheral blood samples (R²=0.79), suggesting its potential suitability for less-invasive clonal monitoring.

**Conclusions:**

In conclusion, we present an adaptable tool for quantification of chromosomal aberrations, particularly in problematic samples, which should be easily applicable to further tumour entities.

## Introduction

The acquisition of cytogenetic abnormalities is a frequent event among various clonal malignant disorders. In particular, such lesions can be detected in 30%–50% of the bone marrow (BM) of patients with myelodysplastic syndromes (MDS).^1^ Interstitial deletions of the long arm of chromosome 5 (del(5q)) are the most commonly observed aberrations found in approximately 30% of affected patients with chromosomal abnormalities.^2^

The current gold standard for karyotypic stratification of patients with MDS is represented by chromosomal banding analysis (metaphase cytogenetics (MC)), which provides an overview of the whole karyotype without prior knowledge.^3^ However, major drawbacks of this method are the requirement for viable cells capable of cell division and a potential bias in the cytogenetic profile due to highly proliferative subclones. Moreover, despite using optimised protocols, MC analyses fail in approximately 5% of cases.^2 4^

Fluorescence in situ hybridization (FISH) uses fluorescently labelled DNA probes for detection of chromosomal alterations in interphase cells. However, the method requires prospectively archived cells for analysis and is relatively expensive and labour-intensive.^5 6^ Alternatively, SNP microarrays solely require genomic DNA and are valuable tools for de novo genome-wide screening of (submicroscopic) copy number alterations,^7–9^ but are highly expensive and allow only semi-quantitative characterisation.

In order to overcome the limitations of the described methods, we developed a novel technique based on the assessment of allelic loss at heterozygous short tandem repeat (STR) markers. These represent repetitive DNA motifs consisting of 2–5 nucleotides that are densely distributed throughout the human genome. Consequently, we aimed to establish a quick, inexpensive and robust technique that allows accurate quantification of genomic aberrations from only minute amounts of input DNA.

## Materials and methods

### Patient and control subjects

Diagnostic BM and peripheral blood (PB) aspirates were collected from 135 patients suffering from MDS or secondary acute myeloid leukaemia (AML) in the Department of Hematology and Oncology of the Medical Faculty Mannheim, University of Heidelberg, Germany, during 2009 and 2015 after written informed consent. The use of human material within this study was approved by the local Institutional Review Board. Patients with MDS were subdivided into A) MDS with del(5q) either isolated or with additional cytogenetic aberrations (n=86, mean age 68 years (range 44–91))and B) MDS with confirmed absence of del(5q) (n=49, mean age 70 years (range 20–90)) by means of MC following the guidelines of the International System for Human Cytogenetic Nomenclature (ISCN).^10^ As non-del(5q) controls (n=125), we isolated PB specimen from healthy donors and cord blood. In addition, serial chronological BM samples (n=95) following treatment with lenalidomide (LEN) were analysed from n=40 del(5q) patients, who were enrolled within the LEMON-5 clinical trial.^11^

### Sample preparation

BM or PB aspirates were subjected to Ficoll density gradient centrifugation (GE Healthcare, Munich, Germany) for isolation of mononuclear cells. For germline correction, mesenchymal stromal cells (MSCs) for n=47 patients were expanded in vitro as described previously.^12^ Fluorescence activated cell sorting (FACS) analysis of MSCs demonstrated high purities and absence of residual haematopoietic cells. DNA was isolated using the Allprep DNA/RNA Mini Kit (Qiagen, Hilden, Germany) according to manufacturer’s instructions.

### Cytogenetics

For cytogenetic confirmation of del(5q), MC analyses were carried out as reported previously.^13^ For quantitative interrogation, 5×10^4^ cells were fixed in methanol:acetic acid (3:1) and interphase FISH was carried out with probes targeting *EGR1* (5q31) and RPS14 (5q33) (MetaSystems, Altlußheim, Germany). Subsequently, 200 cells were analysed on a MetaSystems scanning system (Metafer4, MetaCyte). Cytogenetic analyses were blinded to the results of the paired STR test.

### Polymerase chain reaction

For multiplex-PCR amplification of 12 separate STR loci within the region of del(5q), the fluorochromes FAM, HEX and TAMRA were used as 5’ forward primer labels and for each fluorochrome four non-overlapping STR-flanking PCR products with varying average sizes ranging from ~100 to ~400 bp were generated (see online supplementary table S1).

10.1136/jmedgenet-2017-104528.supp1 Supplementary file

In a volume of 25 µl PCR reactions were carried out using the Type-it Microsatellite PCR Kit (Qiagen) with 5 pmol of each primer and 10 ng DNA unless stated otherwise. The following PCR conditions were used: 95°C 5 min, 29 cycles of 95°C for 30 s, 57°C for 3 min, 72°C for 30 s and a final elongation of 60 min at 60°C for complete extension of ‘A’-overhangs. PCR products were subsequently diluted 1:100 with laboratory grade H_2_O. A further 1:10 dilution was performed in Hi-Di formamide (Life Technologies, Carlsbad, California, USA) containing 1.5% GeneScan 500XL ROX Standard (Life Technologies) followed by 5 min denaturation at 95°C; 10 µl of the final dilution were loaded into an ABI XL3130 system and subjected to capillary electrophoresis.

### Calculation of del(5q) cell fractions

Blinded peak calling was carried out on *fsa*-files using Genemapper V.4.0 software (Life Technologies) using bin sets derived from multiple healthy donors. This was followed by manual verification to ensure that peak patterns resembled the germline pattern derived from concomitantly analysed patient-matched MSC samples. If no germline control for profiling was available, only peaks were taken into account that could confidently be differentiated from PCR-stutter. Markers for which the non-deleted allele peak exhibited an area under the curve (AUC) below 2500 (insufficient PCR product) or above 25 000 fluorescence units (signal saturation) were discarded from subsequent analysis. All data were assessed retrospectively for collected specimen.

For correction of PCR-stutter that might overlap with the shorter PCR fragment (peak1), we screened all control samples for homozygous STR loci. For homozygous markers with *R* repeats, the relative proportion of stutter peaks at positions *R-1*, *R-2*, *R-3* and *R-4* relative to the index peak (peak2) were determined. By averaging this proportion, a marker-specific correction factor(CF_i_) was derived specifically for each combination of stutter position (from *R-1* to *R-4*) and corresponding absolute fragment length (total number of repeats *‘R’*).

If in a heterozygous sample the shorter allele overlapped with such a stutter peak at position *R-1* to *R-4,* the lower PCR peak was corrected as follows:

$$peak1.corr=peak1-(CF_{i}\times peak2)$$

Subsequently, we calculated the degree of skewing for the superior allele, p_sup_, with AUC peak values from corresponding normal (n) and tumour (t) samples:

$$p_{sup}=1-\left(\frac{\frac{\frac{peak2\;t}{peak1.CORR\;t+peak2\;t}}{\frac{peak2\;n}{peak1.CORR\;n+peak2\;n}}}{\frac{\frac{peak2\;t}{peak1.CORR\;t+peak2\;t}}{\frac{peak2\;n}{peak1.CORR\;n+peak2\;n}}+\frac{\frac{peak1.CORR\;t}{peak1.CORR\;t+peak2\;t}}{\frac{peak1.CORR\;n}{peak1.CORR\;n+peak2\;n}}}\right)$$

The p_sup_ was subsequently translated into the proportion of cells carrying del(5q):

$$delcell[\%]=1-\frac{0.5-|(0.5-p_{sup})|}{0.5+|(0.5-p_{sup}|}$$

These calculations were performed for each marker individually and subsequently the mean value derived from all informative markers was used for further analysis.

The frequency of deleted cells by quantification of skewing of heterozygous SNPs derived from high throughput sequencing was determined as previously reported.^14^

Analyses were performed in Microsoft Excel (V.14.0.7153) as well as custom scripts using *R*, V.3.1.3.^15^ An excel file for calculation of del(5q) frequencies from ‘Genemapper’ software derived peak raw data is provided in online supplementary table S2.

10.1136/jmedgenet-2017-104528.supp2 Supplementary table S2

### Statistical analysis

Statistical analyses were carried out using Pearson’s correlation for standard curve, quantitative correlation for FISH or method comparisons. For two group comparisons, Student’s t-test was used. Analyses were performed using Graphpad Prism 6 (GraphPad Software, La Jolla, California, USA) or *R*. Diagnostic accuracy was determined using the R package pROC V.1.8^16^ with default settings.

## Results

### Simultaneous analysis of 12 STR markers allows accurate estimation of del(5q) burden

**Figure 1:**
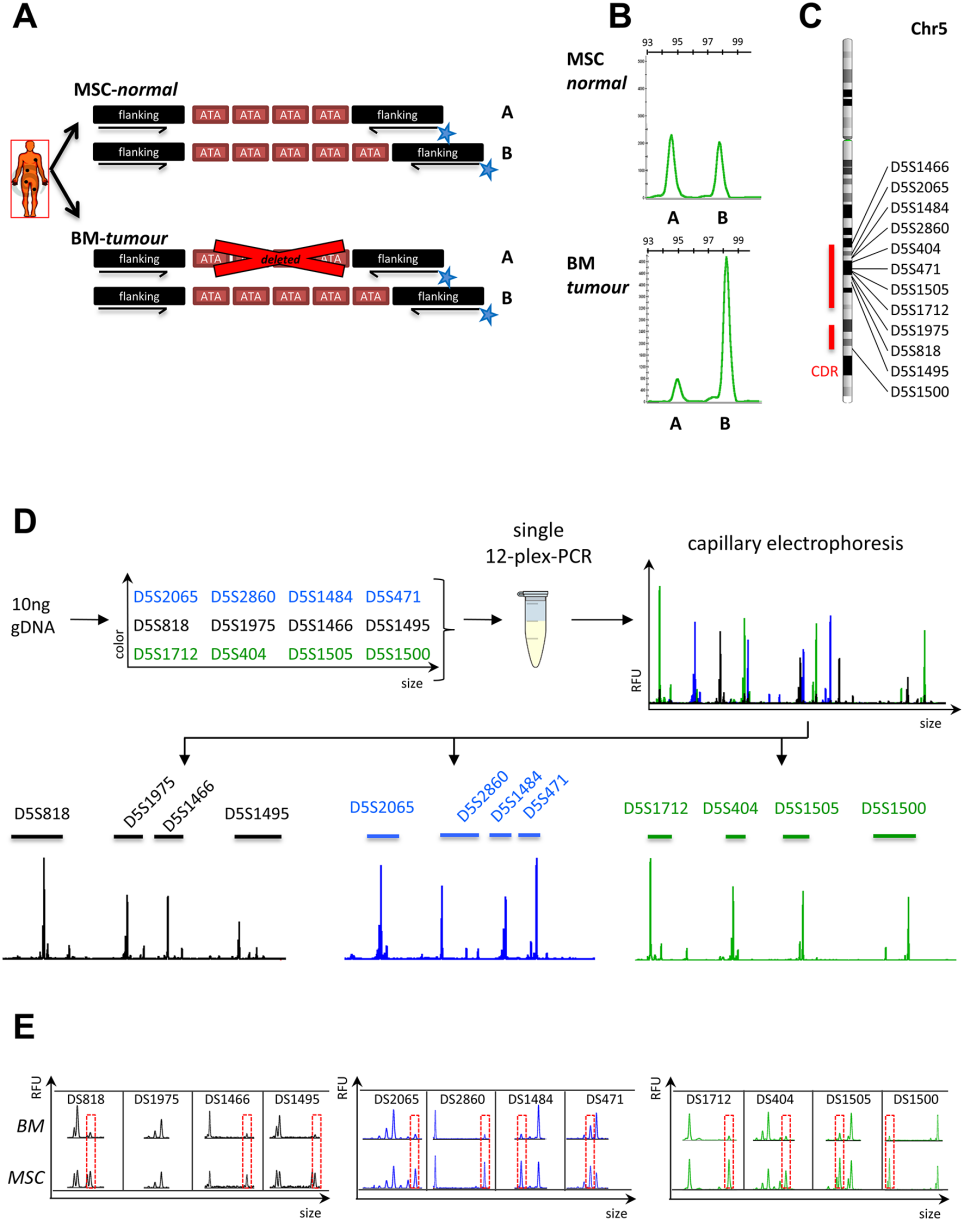
(A) Depiction of fluorochrome primer design for interrogation of allele-specific loss at short tandem repeat (STR) loci located in a deleted chromosomal region. (B) Exemplary peak profile of STR marker D5S1712 for mesenchymal stromal cells (MSCs) demonstrating equal amplification of both alleles and for the bone marrow (BM) sample from the same patient showing loss of allele ‘A’ due to genomic deletion of this particular locus. (C) Chromosomal distribution of selected STR markers among the long arm of chromosome 5 commonly deleted region (CDR) according to^17 18^ (D) experimental setup for multiplex-PCR amplification of 12 selected STR markers and subsequent separation of marker allele profiles for quantification of individual allelic ratios. (E) STR-marker profiles from an exemplary patient sample showed consistently reduced peak intensities of PCR fragments derived from the deleted allele for heterozygous loci (red boxes). Only D5S1975 appeared to be homozygous and therefore uninformative. RFU, relative fluorescence unit.

In order to integrate the concept of STR-based analysis with quantitative copy number evaluation, we designed fluorochrome-labelled PCR primers flanking the repetitive regions of STR loci within the del(5q) region (see online supplementary table S1). Using these primers for PCR amplification of heterozygous STR loci should ideally result in equal amplification of two alleles in germline samples (eg, MSCs), but strongly reduced amplification from the deleted allele in the tumour sample, for example, BM (figure 1A). Indeed, exemplary capillary electrophoresis reliably confirmed skewing of allele-specific PCR-amplicon intensities in the tumour but not corresponding germline sample (figure 1B), thus demonstrating the validity of this approach.

In order to generate robust estimates of del(5q) cell frequencies from DNA, we developed a multiplex-PCR assay that interrogates 12 independent STR markers, distributed along the commonly deleted region within del(5q),^17 18^ in a single reaction (figure 1C). By implementing three fluorochromes and non-overlapping PCR amplicon sizes in the PCR design (see online supplementary table S1), each individual marker could be reliably evaluated (figure 1D and E).

**Figure 2:**
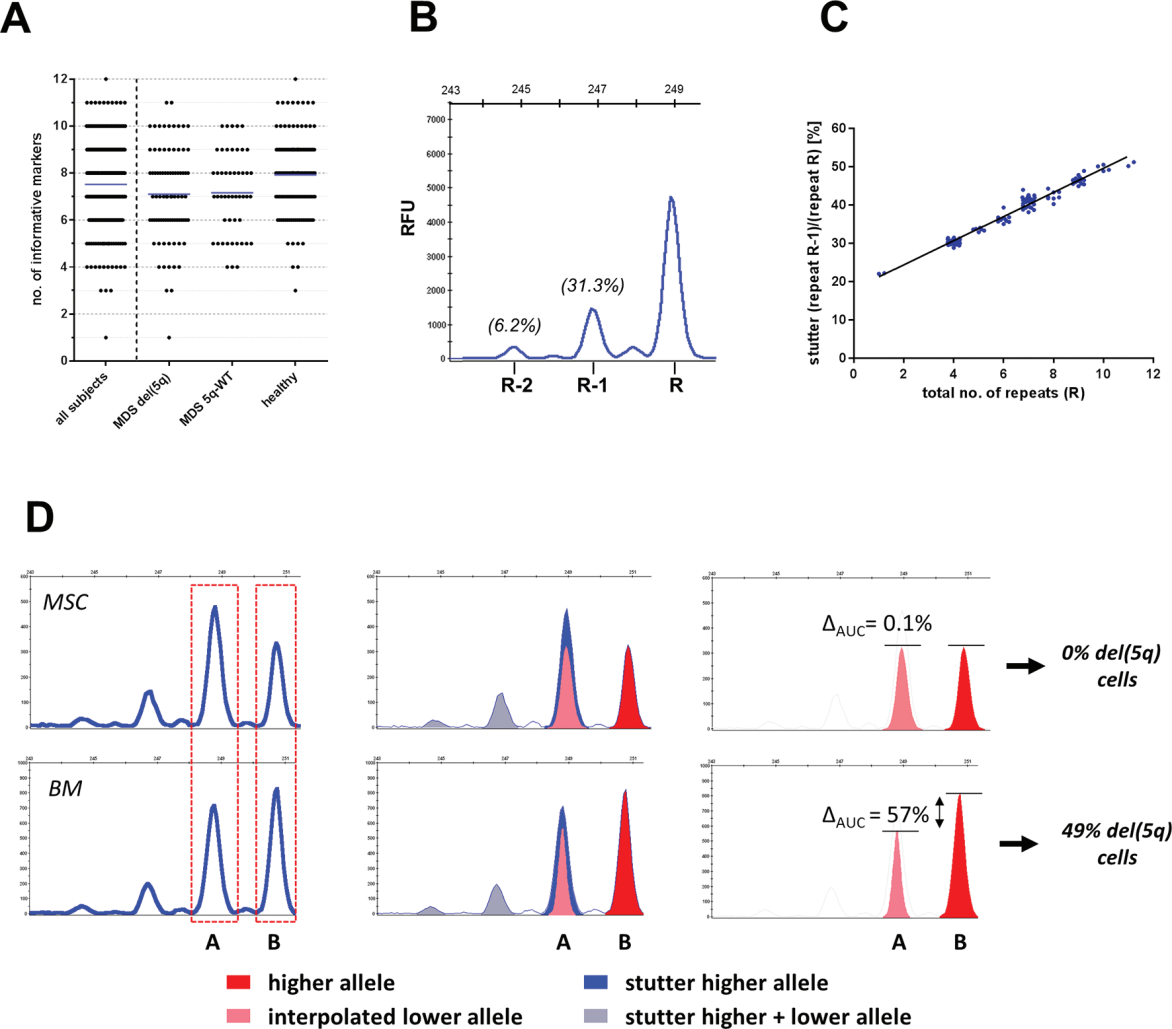
(A) Distribution of the number of informative (heterozygous) short tandem repeat (STR) markers for the entire study cohort and related subgroups. (B) Exemplary peak pattern for homozygous STR marker D5S417 showing its allelic peak at 248 bp and the corresponding PCR-stutter peaks at 246 and 244 bp. (C) Proportion of the ‘R-1’ stutter peak as a function of the total number of repeats exemplarily shown for D5S471. (D) Schematic depiction of correction for PCR-stutter contribution to a coinciding lower allele peak ‘A’ and subsequent translation into fractions of del(5q) cells from corrected allele ratios.

In total, n=1142 samples were analysed from n=260 individuals. By analysing 12 STR markers, we identified on average 7.5 markers (range 1–12) per subject that were heterozygous and therefore informative (figure 2A). Of note, none of the investigated samples exhibited microsatellite instability. For each marker separately, the degree of allelic skewing was translated into frequencies of cells carrying del(5q) using equations outlined in the ‘Materials and methods’ section. Subsequently, results from all informative markers were averaged to obtain the proportion of del(5q) cells for the respective sample.

A total of 259 out of 260 individuals (99.6%) harboured ≥3 informative markers for del(5q) estimation. In 93% (80/86) of del(5q) cases, we observed homogeneous skewing for all interrogated STR markers, suggesting that all markers were located in the deleted region, which underlines the suitability of the selected STR marker based on a large cohort of del(5q) patients. In the remaining six cases, some markers were located outside the patient-specific deleted region. However, all six individuals harboured informative markers within the deleted region that could be used to quantify del(5q). Absolute marker intensities were homogeneous among all 12 loci indicating highly similar PCR amplification efficacies. Moreover, comparison of del(5q) cell frequencies obtained by any two marker combinations resulted in strong congruence (mean R²=0.92, range: 0.77–0.97, see online supplementary figure S1).

### PCR-stutter correction

**Figure 3:**
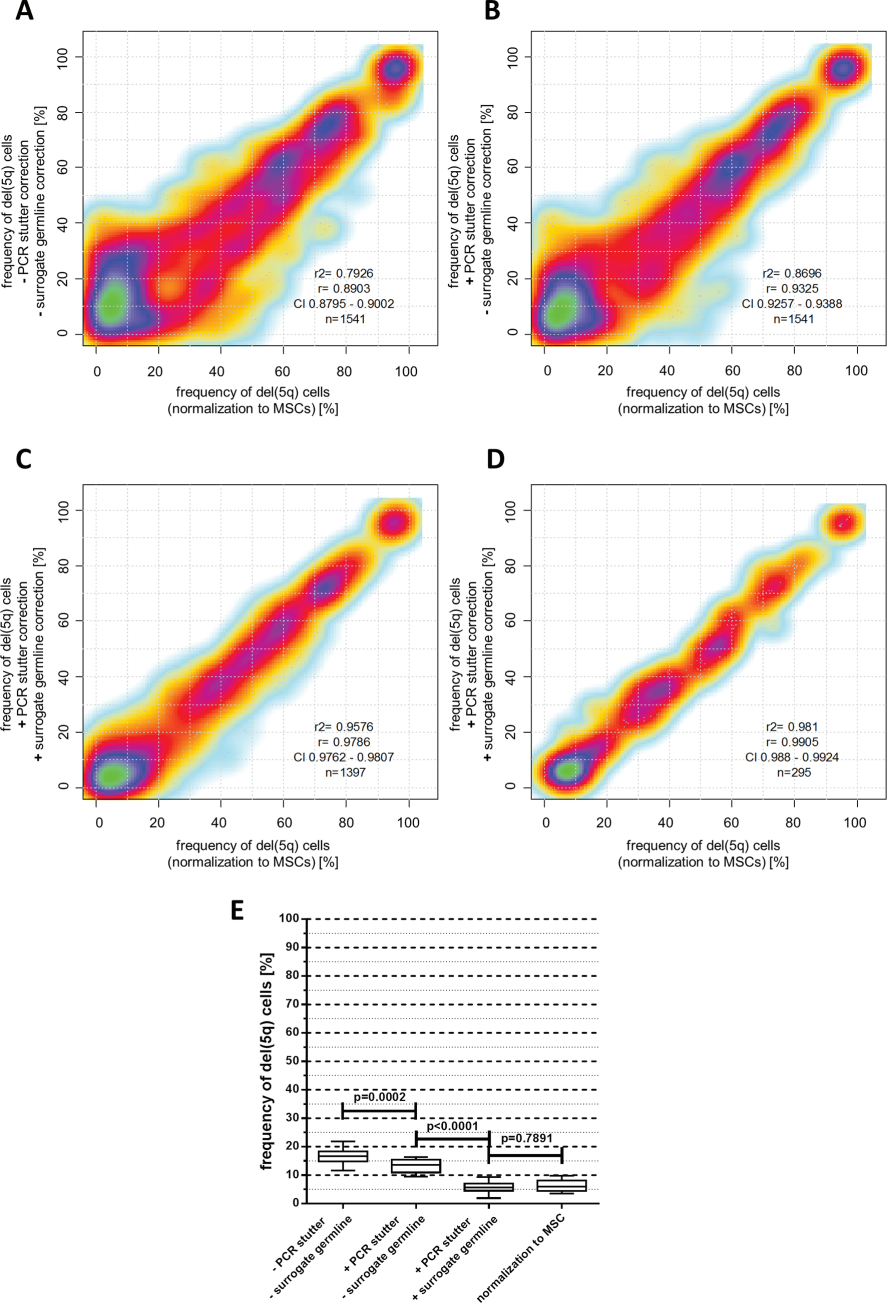
(A–C) Correlation of del(5q) frequencies calculated from individual short tandem repeat (STR) markers in haematopoietic cells normalised to matched mesenchymal stromal cell (MSC) germline samples (x-axis) and their corresponding del(5q) fractions (without MSC normalisation) calculated either with or without PCR-stutter and ‘surrogate’ germline correction (y-axis). (D) Comparison of average del(5q) fractions derived from all informative markers for n=295 haematopoietic samples either normalised to a corresponding MSC sample (x-axis) or corrected for PCR-stutter and normalised to ‘surrogate’ germline controls (y-axis). (E) Reduction of offset del(5q) frequency measurements in 5q-wildtype (5q-WT) samples depending on PCR-stutter and surrogate germline correction (whiskers represent 10%–90% data range).

For some STR markers, particularly those consisting of 2 bp repeats, allele peaks were accompanied by additional peaks that were exactly 1–4 repeat lengths shorter. This phenomenon of ‘PCR-stutter’ is well known^19^ (figure 2B). Because stutter peaks can potentially overlap with the shorter allele peak, we quantified their relative size in homozygous markers at position (R-1) to (R-4) for all observable alleles with R repeats in a cohort of n=125 healthy controls. On average, 10 individuals (range 1–61) were homozygous for any observed absolute marker fragment size. Strong correlations between relative stutter size and total number of repeats were found for several markers (figure 2C). Exemplarily, for a patient sample with heterozygous locus D5S471 the (R-1) stutter peak from the higher allele (251 bp) overlapped with the lower allele (249 bp) (figure 2D) resulting in a markedly increased lower allele peak in the MSC sample. Consequently, this pattern would theoretically indicate the presence of del(5q) cells in the germline sample. However, six healthy individuals showed an average relative stutter of 33.5% at the (R-1) position for the 251 bp allele of D5S471. After subtracting this proportion from the lower allele, the relative differences between lower and higher allele peak sizes changed to 0.1% for the germline control (<1% del(5q) cells) and 57% for the BM sample (49% del(5q) cells). This preprocessing strategy considerably improved the accuracy of del(5q) estimation from 1541 individual marker observations from R²=0.79 to R²=0.87 (figure 3A and B).

### Determination of ‘surrogate’ germline control profiles

During our analysis of heterozygous markers in MSC germline controls, we observed slightly preferential amplification of the shorter allele resulting in a lower to higher peak ratio of >1. This phenomenon of ‘allelic imbalance’ was variable for each locus and dependent on the difference of fragment sizes between the alleles as well as the absolute length of each fragment. Fortunately, this bias could be effectively compensated by germline normalisation.

However, routine clinical acquisition of germline material is often impracticable and for archived patient DNA samples such controls are usually not available. In order to improve the accuracy of del(5q) quantification for samples without controls, we aimed to establish a comprehensive database of ‘surrogate’ germline profiles for the most frequently observed STR allele patterns. This was achieved by averaging the lower to higher allele peak ratio for every heterozygous marker allele combination observed in n=125 healthy donors. In total, a median number of 6 (range 1–69) observations for 190 unique combinations could be generated. The observed allele ratio imbalance for any particular allele combination was highly uniform among individuals (mean SD=8%).

Finally, for n=295 samples with available germline controls, we calculated fractions of del(5q) cells for every informative marker (n=1397) via normalisation 1) with our surrogate germline database and 2) using patient-matched MSC germline counterparts. Both approaches resulted in high concordance for del(5q) estimates from individual markers (R²=0.96, figure 3C) and even more robust concordance for averaged *per sample* frequencies from all informative markers (R²=0.98, figure 3D). Moreover, ‘surrogate’ germline correction considerably reduced the measurable offset for del(5q) frequency estimation in 5q-wildtype (5q-WT) cases from 16.9% to 6.2% (p<0.0001, figure 3E), reflecting the assay’s low background noise level.

### Validation of STR-based del(5q) quantification

**Figure 4:**
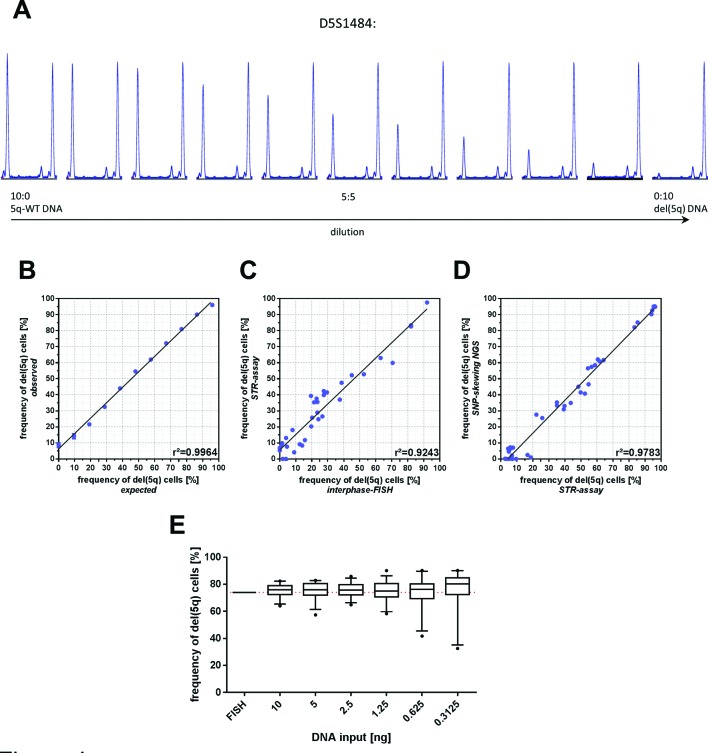
(A) Exemplary peak patterns for marker D5S1484 depicting the gradual increase of allele skewing in a serial dilution series. (B) Correlation of expected and observed del(5q) frequencies derived from defined mixed ratios of DNA carrying del(5q) (bone marrow) and 5q-wildtype (5q-WT) alleles (mesenchymal stromal cell (MSC)). Error bars depict SD from triplicate reactions. (C) Comparison of the frequency of del(5q) positive cells determined by concomitant interphase FISH and short tandem repeat (STR)-assay analysis. (D) Comparison of the frequency of del(5q) positive cells determined by next-generation sequencing-based SNP skewing or STR-assay analysis. (E) Impact of decreasing input amounts of DNA to the STR-PCR reaction. Each box plot reflects the results of individual markers obtained from three replicate reactions. Whiskers represent 5%–95% data range.

In order to determine the accuracy of our PCR-based assay, we performed a serial dilution series with defined ratios of del(5q) and 5q-WT DNA obtained from the same individual. Correlation of expected and detected proportions of del(5q) cells revealed highly concordant results (R²>0.99, figure 4A and B). Of note, paired analysis using our PCR-based assay and interphase-FISH from n=34 samples resulted in a strong correlation of measured del(5q) cell frequencies (R²=0.92, figure 4C) validating the suitability of STR-based quantification of del(5q) burdens. Moreover, this PCR-based method also showed high quantitative correlation with skewing of heterozygous SNPs for n=37 paired samples (R²=0.98, figure 4D).

### Assay reproducibility

To address the assay’s reproducibility, we performed replicate analysis for n=385 samples and found an overall mean SD of 1.7% for del(5q) cell frequencies that were calculated from the average of all informative markers (see online supplementary table S2). Moreover, the reproducibility of individual markers was also highly comparable and ranged from 2.1% (D5S1495) to 4.6% (D5S1466).

### PCR input

As this assay was designed to quantify del(5q) frequencies from samples with only limited available DNA, we aimed to determine its robustness with decreasing amounts of input DNA. Interphase-FISH analysis of a selected BM sample revealed 74% del(5q) positive cells (148/200 cells) (figure 4E). Consistently, STR-based quantification using 10 ng DNA isolated from the same specimen resulted in 75.3% (95% CI 73.7% to 76.9%) cells carrying del(5q). By serially decreasing the DNA input down to 0.3125 ng (equivalent to ~50 cells), the mean proportion of del(5q) positive cells for this DNA amount was 75.5% (95% CI 70.28% to 80.64%, figure 4E), which did not vary from 10 ng input (p=0.94) underlining the suitability of this assay even with ultra-low DNA input.

### Monitoring of uniparental disomy and additional genomic lesions

**Figure 5:**
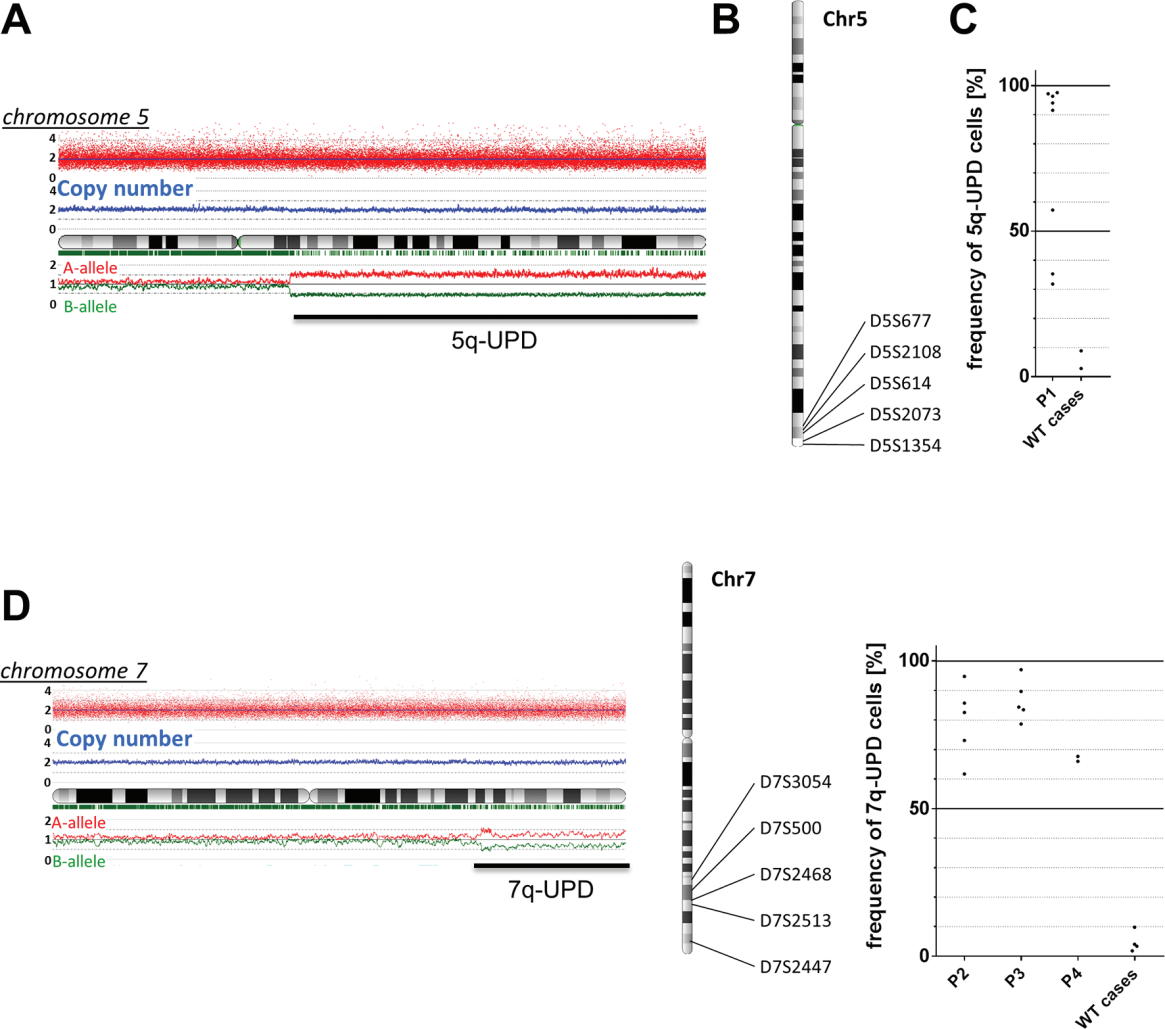
(A) Affymetrix SNP V.6.0 array analysed using CNAG V.3.0 software shows a telomeric uniparental disomy (UPD) at chromosome 5q. While the data indicate that a copy number of 2 is maintained throughout all chromosomal regions, telomeric loss of one allele can be detected on the long arm of the chromosome. (B) Chromosomal distribution of selected STR markers specific for the UPD region on chromosome 5q. (C) Quantification of the fraction of cells carrying 5qUPD in multiple samples from this patient (P1) and wildtype (WT) cases. (D) Left: SNP V.6.0 array shows a telomeric UPD at chromosome 7q. Middle: chromosomal distribution of selected STR markers for the UPD region on chromosome 7q surrounding the EZH2 locus. Right: quantification of the fraction of cells carrying the UPD for n=3 patients (P2–P4) and WT cases.

Loss of heterozygosity at STR-loci can result from genomic deletions and from acquired uniparental disomy (UPD). As such, as an important advantage compared with FISH and MC, our established assay additionally provides quantitative information about UPDs. In a recent study, this approach allowed us to identify a patient with MDS with a clone carrying the del(5q) lesion that further evolved into telomeric UPD of 5q^14^ as validated by SNP-array analysis (figure 5A). For accurate distinction between del(5q) and 5q-UPD, we established a separate set of telomeric STR assays (figure 5B). This allowed us to reliably quantify the burden of cells carrying 5q-UPD (figure 5C), but also 7q-UPD (figure 5D).

Exemplarily, expanding STR-based analysis to additional chromosomal deletions allowed precise quantification of del(9q) and del(20q) aberrations (see online supplementary figure S2) indicating the assay’s ability for interrogating a diverse set of genomic aberrations.

### Suitability to monitor clonal burden under therapy

**Figure 6:**
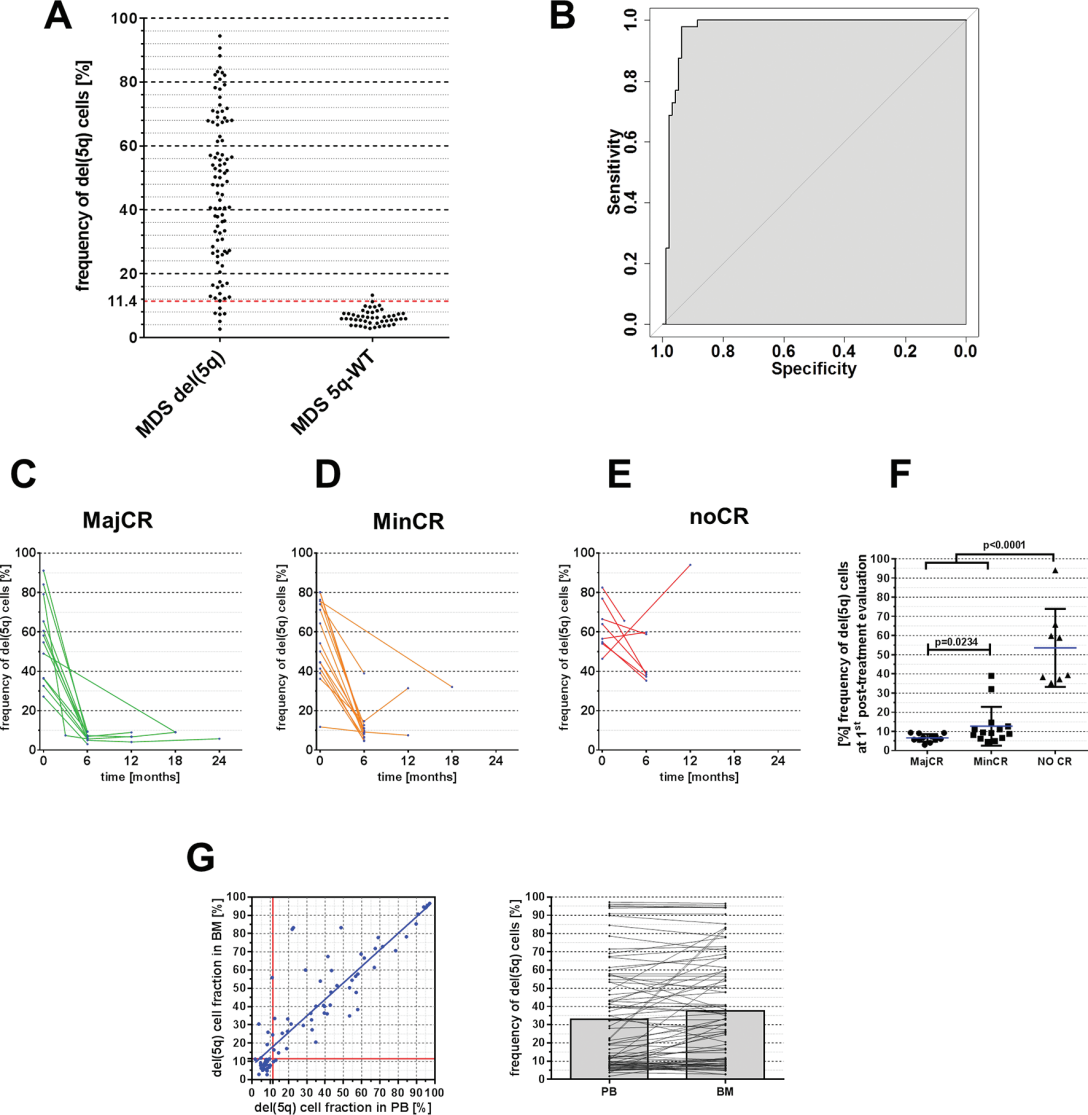
(A) Distribution of observed del(5q) fractions as determined via short tandem repeat (STR)-based quantification for patients with del(5q) and 5q-WT. Red dotted line at 11.4% indicates chosen diagnostic threshold from receiver operating characteristic (ROC) analysis. (B) ROC analysis illustrating the sensitivity and specificity of STR-based del(5q) stratification in patients with MDS with del(5q) and 5q-WT. (C–E) STR-based quantification of del(5q) burden in bone marrow (BM) following treatment with lenalidomide (LEN) subdivided to cytogenetic response groups. MajCR, major cytogenetic response; MinCR, minor cytogenetic response; noCR, no cytogenetic response. (F) Summary of del(5q) frequencies on patients’ first post-LEN BM examination according to observed clinical response. (G) Correlation of del(5q) cell fractions using STR-based assessment in matched peripheral blood (PB) and BM samples.

To evaluate the utility of STR-based del(5q) measurement as a diagnostic tool in a clinical setting, we first compared quantitative STR results of BM samples from patients with MDS who were cytogenetically confirmed del(5q) positive (n=137) or 5q-WT (n=44) as defined by ISCN criteria.^10^ Among del(5q) patients, we observed variable clonal burdens of del(5q) with clone sizes ranging from 2.7% to 94.4% del(5q) cells in BM (figure 6A). However, in the 5q-WT group we also found minor imbalances eventually translating into a mean proportion of 6.4% del(5q) positive cells which is considered as non-significant background. Thus, we aimed to determine our assay’s diagnostic accuracy for classification of patients into del(5q) or 5q-WT cases and performed receiver operating characteristic analysis, which revealed an AUC of 0.97 (95% CI 0.94 to 0.99) (figure 6B). The optimal diagnostic cut-off, defined as the threshold providing the maximum distance to the diagonal identity line,^20^ was found to be 11.4% del(5q) cells resulting in a sensitivity and specificity of 93.8% and 97.9% (positive predictive value: 98.9%, negative predictive value: 88.7%), respectively (see online Supplementary material).

Next, we sought to assess the utility of STR-based quantification for chronological patient monitoring. For this, serial samples from a cohort of n=40 patients, who were enrolled in the LEMON-5 trial from the German MDS study group were monitored for changes in clonal del(5q) burden upon treatment with LEN. To put these data into relation with established response criteria,^21^ centralised cytogenetic follow-up analysis was compared with STR-based del(5q) quantification. While cytogenetic analyses failed (ie, metaphase failure) in 5/91 (5.5%) cases, our STR-PCR assay successfully generated del(5q) estimates in all interrogated samples. Among 12 patients, who achieved major cytogenetic response, determined by complete absence of aberrant metaphases, the mean proportion of cells carrying del(5q) was 6.7% (range 3%–10%) (figure 6C), which was below the previously defined detection threshold and therefore has to be classified as ‘negative’ in agreement with MC results. In addition, this proportion was significantly lower as compared with n=15 patients achieving only minor cytogenetic response, defined as ≥50% reduction of aberrant metaphases (figure 6D and F, mean: 12.7% del(5q) cell frequency, range 5%–39%, p=0.023). Notably, none of the patients without cytogenetic response (n=8) showed a reduction below 38% del(5q) cells after treatment with LEN (figure 6E). In summary, these data suggest that STR-based quantification of del(5q) represents a reliable approach for monitoring del(5q) clonal burden during clinical follow-up.

Finally, we tested the applicability of the assay for del(5q) monitoring in PB cells, which are generally easier to collect than BM aspirates. Indeed, frequencies of del(5q) positive cells in n=83 matched PB and BM samples revealed a robust concordance (R²=0.79, figure 6G) despite slightly lower burden in PB cells.

## Discussion

Using a multiplex-PCR assay for simultaneous measurement of 12 independent STR markers, we developed a highly adaptable tool for precise quantification of chromosomal lesions. By analysing a large collection of n=1142 samples, our DNA-based assay provided accurate assessment of cell frequencies carrying del(5q), as an exemplary lesion, which was confirmed via replicate analysis and correlation with paired interphase-FISH results. Importantly, our assay does not require dividing or fixated cells and therefore is highly suitable for copy number quantification in samples for which only residual DNA is available. While microarray or qPCR-based methods could also be used for targeted interrogation of chromosomal lesions from DNA,^22 23^ they are costly and provide only semi-quantitative results. Additionally, this assay resembles quantitative copy number quantification of current next-generation sequencing approaches that quantify allelic skewing of heterozygous SNPs, which are, however relatively expensive and require prior knowledge of the individual’s SNP genotypes. With approximately US$2 per reaction, multiplex-STR analysis is relatively inexpensive. With only 1 hour hands-on time for measurement of 96 DNA samples, the procedure generates results in <24 hours and is easily scalable for lower sample throughput.

In forensic analyses, STR markers represent a key element for genotyping of individuals due to their high rate of heterozygosity and therefore informativity.^24–26^ In line with this, all of our 260 investigated individuals harboured informative markers within the investigated region of del(5q). Usually, analysis of STR markers suffers from PCR-related side effects such as PCR-stutter and small variations in allele-specific PCR efficiencies (allelic imbalances), which even occur in more stable trinucleotide to pentanucleotide repeats. Most of these artefacts can be corrected for by using patient-matched germline samples, for example, MSCs. However, because such controls are often unavailable for archival specimen or in a routine clinical setting, we developed an effective compensation matrix by integrating data from a large control cohort. With this, ‘surrogate’ germline profiles and PCR-stutter corrections could be established for almost every observed marker allele combination. Notably, del(5q) quantification using either ‘surrogate’ or patient-matched MSC germline correction demonstrated a strong correlation (R²>0.98) across n=295 individual samples. For convenience, we provide an excel sheet (see online Supplementary table S2) that can be easily employed for calculation of del(5q) frequencies from raw data. By implementing our comprehensive correction database in this file, all raw values are fully automatically corrected without need for manual intervention and subsequently translated into the fraction of del(5q) cells.

From specimens with low cell yield and quality, such as rare FACS-sorted fractions, BM smears or colony-forming units, it is particularly challenging to obtain accurate and quantitative copy number data. Importantly, due to the small amplicon sizes our assay should also be applicable for investigation of fragmented DNA from formalin-fixed archived specimen. Moreover, our finding of high accuracy even with ultra-low DNA input equivalent to 50 cells suggests that STR-based analysis is highly applicable for copy number interrogation from such problematic samples. In this context, our assay has recently been successfully used to decipher the chronological relationship between chromosomal and point or small insertion/deletion mutations by investigating a large number of samples of low cellular abundance.^14^

Diagnosis of del(5q) has important implications for stratification of patients with MDS. Compared with MC, STR-based del(5q) quantification provided high diagnostic accuracy for del(5q) assessment. In addition, the method reliably monitored del(5q) clonal burden during treatment with LEN suggesting its applicability as an alternative DNA-based technique for cytogenetic clone-size evaluation. Importantly, copy number neutral lesions such as acquired UPD can also be identified using this assay, representing a significant advantage over currently used standard diagnostic procedures.

Previous studies already indicated that copy number aberrations in BM cells can often also be detected in corresponding PB samples.^27–31^ In agreement with this, cross-comparison of STR-based del(5q) quantification in paired BM and PB samples showed reliable correlation, possibly allowing the use of such easily obtainable specimen for clinical clone size monitoring.

In summary, our newly developed DNA-based PCR assay provides an inexpensive tool to obtain quantitative data for a diverse set of chromosomal aberrations, which contain STRs and should be easily applicable to other clonal diseases.
