# Supplementary material for: Accurate quantification of chromosomal lesions via short tandem repeat analysis using minimal amounts of DNA
Source: J Med Genet. 2017 Jun 9;54(9):640–50. doi: 10.1136/jmedgenet-2017-104528 (PMC5574397; doi:10.1136/jmedgenet-2017-104528)
Supplement: Supplementary file [file jmedgenet-2017-104528supp001.pdf]

Supplemental table 1: Marker characteristics.

**Marker**	**Length [bp]**	**Repeat**	**FW-Seq**	**RV-Seq**	**genomic coordinates: [hg38]**	**Flanking feature:**
**D5S1466**	218-239	GATA	[TAM]GTATCAGAACTTCATGTTGTACACC	GGCACCTAGGTTTGTTCTGA	chr5:109683852-109684147	flanking MAN2A1 on 5’
**D5S2065**	109-135	CA	[6FAM]CAGCCTCATTGTTTATTGACAG	AATGGCATAGTTTTGGCTC	chr5:114385482-114385592	intronic KCNN2
**D5S1484**	264-282	ATT	[6FAM]CTACCTATGATGGGGCTGTG	GCTGGGCACTGAAGATTTAG	chr5:115057082-115057360	flanking TRIM36 on 5’
**D5S2860**	188-220	ATCT	[6FAM]TGTTTCTAGCCTGCTTGCTT	AGTGATGGTTCCTCCAATGA	chr5:115706509-115706729	flanking CDO1 on 5’
**D5S404**	180-198	GT	[HEX]CTGGAGATGTAATGCTGTGC	GATCACCACATTCCACCTAAT	chr5:117511504-117511685	intronic LINC00992
**D5S471**	236-248	CA	[6FAM]TTTTCACACATTTTCCCAGC	AAAACTTCATTTACAAAAACAGGAG	chr5:119713364-119713582	flankling FAM170A on 3’
**D5S1505**	243-248	GATA	[HEX]TAAGTGCCAGAGTCTCCCAC	TAAGGCATGTCTCGGAGCTA	chr5:119765951-119766207	flankling FAM170A on 3’
**D5S1712**	88-106	ATA	[HEX]GCTTCCAAGTCAGTCTTTGG	GATTAGATACCAGTCCCTGCC	chr5:121367389-121367495	flanking FTMTon 5’
**D5S1975**	246-260	CA	[TAM]GCTGTGCTGTTTGGCTA	ACCTCATCTTTCGGGCTA	chr5:122789279-122789530	intronic SNX2
**D5S818**	156-157	GATA	[TAM]GGGTGATTTTCCTCTTTGGT	TGATTCCAATCATAGCCACA	chr5:123775491-123775639	flanking CSNK1G3 on 3’
**D5S1495**	383-384	GATA	[TAM]TCCAAAAAATGATTTGAGGC	TAAAAGATGTCTCTCTTTTTGCG	chr5:126014300-126014686	flanking GRAMD3 on 5’
**D5S1500**	353-386	GATA	[HEX]CTCTCTGTGCCCCTCTCTAA	CAGTGAAAATTAATGAAATATGGC	chr5:144839079-144839440	flankling KCTD16 on 3’

Supplemental table 3: S.D. measurements among all or individual markers derived from duplicate analysis.

name	mean S.D. [%]	95% CI
**Overall***	1.7	1.5-1.9
**D5S2065**	2.9	2.2-3.7
**D5S1484**	2.0	1.5-2.4
**D5S2860**	3.1	2.6-3.6
**D5S471**	2.7	2.3-3
**D5S1712**	3.6	3-4.2
**D5S1500**	2.7	2.2-3.2
**D5S404**	2.9	2.4-3.5
**D5S1505**	3.0	2.5-3.6
**D5S1466**	4.6	3.4-5.8
**D5S1975**	2.5	0.2-4.8
**D5S1495**	2.1	0.6-3.5
**D5S818**	2.3	1.5-3

* Overall S.D. for del(5q) quantification was derived from duplicate multiplex reactions after averaging individual marker values

Supplemental table 4: Parameters of diagnostic accuracy for PCR based identification of del(5q).

threshold (95% CI)	4.9	7.2	7.5	7.6	9	11.4*	13.5
sensitivity	0.99 (0.969-1)	0.979 (0.948-1)	0.969 (0.927-1)	0.958 (0.917-0.99)	0.948 (0.895-0.99)	0.938 (0.885-0.979)	0.885 (0.823-0.938)
specificity	0.25 (0.125-0.375)	0.688 (0.562-0.813)	0.729 (0.604-0.854)	0.771 (0.646-0.875)	0.875 (0.771-0.958)	0.979 (0.938-1)	1 (1-1)
PPV	0.725 (0.696-0.76)	0.862 (0.812-0.913)	0.877 (0.826-0.929)	0.893 (0.846-0.939)	0.938 (0.894-0.979)	0.989 (0.938-1)	1 (1-1)
NPV	0.923 (0.75-1)	0.943 (0.86-1)	0.921 (0.837-1)	0.902 (0.814-0.976)	0.894 (0.813-0.977)	0.887 (0.804-0.96)	0.814 (0.739-0.889)

PPV: positive predictive value; NPV: negative predictive value; * optimal diagnostic cut-off.

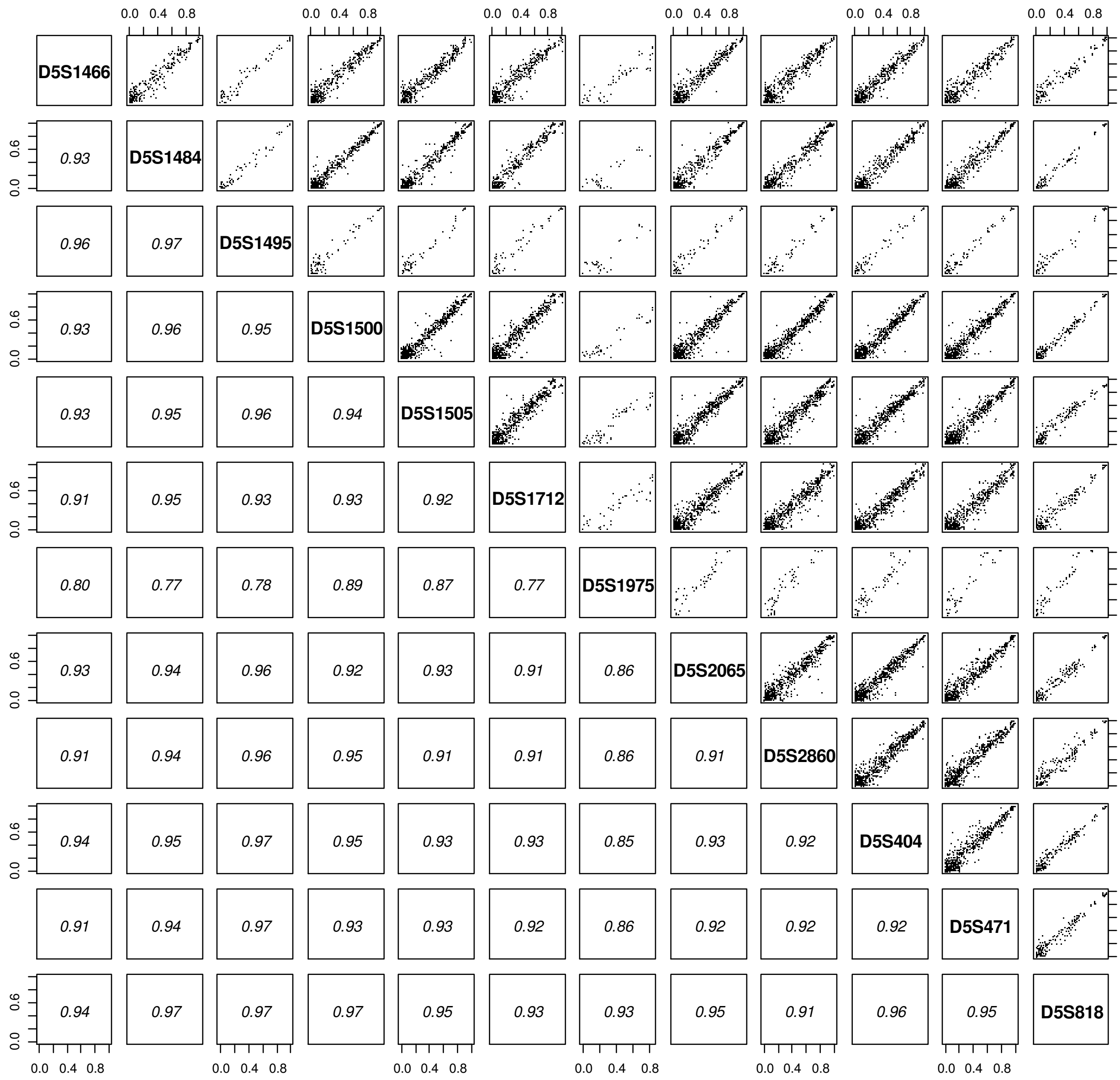

Supplemental Figure 1

Correlation of any possible two-marker comparisons. Numbers indicate R^2.

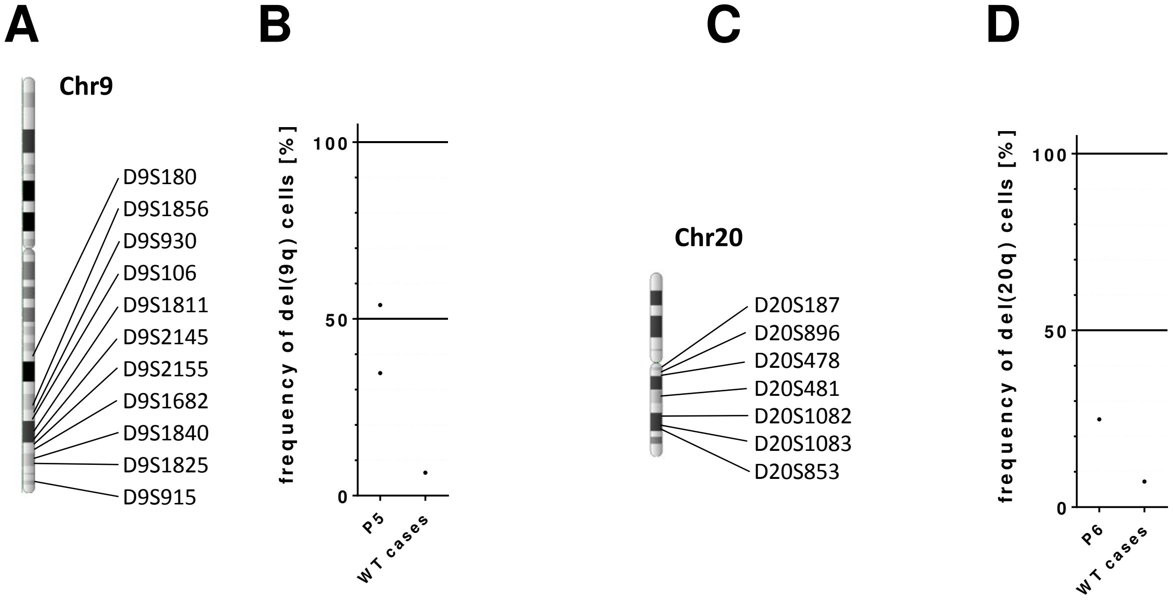

Supplemental Figure 2

A) Chromosomal distribution of selected STR-markers specific for the chromosome 9q (del(9q)) B) Quantification of the fraction of cells carrying del(9q) in patient P5 and a wildtype (WT) case. C) Chromosomal distribution of selected STR-markers specific for the chromosome 20q (del(20q)). D) Quantification of the fraction of cells carrying del(20q) in patient P6 and a wildtype (WT) case.
